# Tétanos associé aux soins: à propos d’un cas

**DOI:** 10.11604/pamj.2016.25.108.9237

**Published:** 2016-10-24

**Authors:** Savadogo Mamoudou

**Affiliations:** 1 Service des Maladies Infectieuses du CHU Yalgado Ouédraogo, Burkina Faso; 2 UFR/SDS Université de Ouagadougou, Burkina Faso

**Keywords:** Tétanos, soins, vaccination, sérothérapie antitétanique, Tetanus, treatment, immunization, antitetanus serum therapy

## Abstract

La prophylaxie antitétanique lors de la prise en charge des blessures, est une stratégie majeure de prévention du tétanos en milieu de soins. Toute défaillance de la prise en charge des blessures expose dangereusement les victimes à la maladie. Nous rapportons un cas de tétanos survenu à la suite d’une blessure frontale prise en charge dans une structure sanitaire sans prophylaxie antitétanique. L’objectif est de rappeler aux cliniciens sur l’importance de cette prophylaxie chez tout blessé non vacciné ou ayant un statut immunitaire douteux. Patient de 52 ans non vacciné contre le tétanos a été admisau CHU YO pour cervicalgie, dysphagie, difficulté à la marche et à l’ouverture de la bouche. Dans ses antécédents il souligne une blessure frontale profonde ayant été suturée sans prophylaxie antitétanique il y a trois semaines environ. L’examen à son admission notaitun trismus lâche, une contracture abdominale, une dysphagie, une température à 36°5Cet une cicatrice au niveau du front mesurant environ 7 cm. Le diagnostic d’un tétanos généralisé stade II à porte d’entrée frontale a été retenu. Sous traitement l’évolution a été favorable et il est sorti de l’hôpital le 18septembre 2015. La prévention du tétanos associé aux soins requiert l’application rigoureuse des mesures d’asepsie, la systématisation de la sérothérapie antitétanique lors de la prise en charge de toute blessure profonde du patient non vacciné ou ayant un statut immunitaire douteux.

## Abstract (en)

Tetanus prophylaxis in routine wound management is a major strategy for tetanus prevention in health care settings. Failed wound management interventions leave patient dangerously exposed to this disease. We report the case of a patient with tetanus occurred after medical treatment for head injury performed in a healthcare facility without appropriate tetanus prophylaxis. This study aims to remind clinicians of the importance of prophylaxis in previously unvaccinated wounded patients or with a doubtful immune status. A 52-year-old patient who had not previously been vaccinated against tetanus was admitted to Yalgado Ouédraogo University Hospital CHU/YO with cervical pain, dysphagia, difficulty walking and opening the mouth. The patient had a personal history of deep wound on his forehead sutured without previous anti-tetanus prophylaxis approximately three weeks before. Physical examination on admission showed loose lockjaw, abdominal spasm, dysphagia, a body temperature of 36,5 °C and a scar on his forehead measuring about 7 cm. The diagnosis of generalized tetanus (stage II) infection occurring after forehead wound was retained. Treatment outcome was favorable and the patient was dismissed on September 18, 2015. The prevention of tetanus associated with an appropriate treatment requires rigorous application of aseptic techniques, systematization of antitetanus serum therapy in the management of previously unvaccinated patients or with a doubtful immune status presenting with a deep wound.

## Introduction

La prophylaxie antitétanique lors de la prise en charge des blessures, est une stratégie majeure de prévention du tétanos en milieu de soins. Toute défaillance de la prise en charge des blessures expose dangereusement les victimes à la maladie. Nous rapportons un cas de tétanos survenu à la suite d’une blessure frontale prise en charge dans une structure sanitaire sans prophylaxie antitétanique. L’objectif est de rappeler aux cliniciens sur l’importance de cette prophylaxie chez tout blessé non vacciné ou ayant un statut immunitaire douteux. Patient de 52 ans non vacciné contre le tétanos a été admisau CHU YO pour cervicalgie, dysphagie, difficulté à la marche et à l’ouverture de la bouche. Dans ses antécédents il souligne une blessure frontale profonde ayant été suturée sans prophylaxie antitétanique il y a trois semaines environ. L’examen à son admission notaitun trismus lâche, une contracture abdominale, une dysphagie, une température à 36°5C et une cicatrice au niveau du front mesurant environ 7 cm. Le diagnostic d’un tétanos généralisé stade II à porte d’entrée frontale a été retenu. Sous traitement l’évolution a été favorable et il est sorti de l’hôpital le 18septembre 2015. La prévention du tétanos associé aux soins requiert l’application rigoureuse des mesures d’asepsie, la systématisation de la sérothérapie antitétanique lors de la prise en charge de toute blessure profonde du patient non vacciné ou ayant un statut immunitaire douteux.

## Patient et observation

Le tétanos est une maladie infectieuse qui demeure un problème majeur de santé publique dans les pays en voie de développement malgré l’existence d’un vaccin efficace, disponible et intégré dans les programmes élargis de vaccination (PEV) [1]. Sa fréquence est variable selon les pays. En Afrique son taux de mortalité varie de 20 à 90% [1–4]. Au Burkina Faso, l’effort de la vaccination a permis de réduire considérablement l’incidence de la maladie avec une tendance à l’éradication du tétanos néonatal. Mais des cas sporadiques survenant chez des sujets non vaccinés sont toujours enregistrés dans les formations sanitaires. Le tétanos de l’adulte est dû souvent à des plaies souillées, négligéesou mal prises en charge. Sa prévention repose sur la vaccination et la sérothérapie antitétanique, associés à l’application d’une asepsie rigoureuse lors de la prise en charge de toute plaie survenant chez le sujet non vacciné ou ayant un état vaccinal douteux. Toute défaillance de la prise en charge des blessures, expose les victimes à la maladie. Nous rapportons un cas de tétanos survenu à la suite d’une insuffisance de la prise en charge d’une blessure avec pour objectif de rappeler les règles élémentaires deprévention du tétanos lors de la prise en charge de toute blessure.

## Discussion

Le tétanos est une toxi-infection aiguë due aux exotoxines protéiques neurotropes produites par *Clostridium tetani*qui est une bactérie anaérobie stricte, tellurique et ubiquitaire. Pour la prévention de cette affection, il existe un vaccin dont l’efficacité et l’innocuité ne souffrent d’aucune contestation.Malgré la mise en œuvre du programme élargi de vaccination sur recommandation de l’OMS, le tétanos reste un problème de santé publique dans les pays en voie de développement [5–7]. Au Burkina Faso, si des efforts sont faits pour l’éradication du tétanos néonatal et pour l’immunisation des nourrissons, il n’en est pas de même pour le reste dela population. La prédominance masculine de la maladie a été suffisamment documentée [7, 8] et semble être en rapport avec la mise œuvre d’une politique d’éradication du tétanos néonatal à travers l’organisation de campagnes répétées de vaccination antitétaniques ciblant les femmes en âge de procréer. Après l’immunisation vaccinale antitétanique de la petite enfance dans le cadre du programme élargi de vaccination, aucune politique de rappels vaccinaux n’est mise en place pour la promotion de la lutte contre le tétanos chez les adultes [7], les exposant ainsi à cette maladie en cas blessure. Une plaie suturéesans sérothérapie avait été la porte d’entrée du tétanos. Dans la série d’Aba et al à Abidjan, les sutures représentaient 55% des portes d’entrée du tétanos et seulement 5% des patients avaient bénéficié d’une sérothérapie antitétanique [3]. Ce taux était moindre dans la série de Traoré et al en Guinée Conakry [8]. La porte d’entrée était céphalique dans notre cas contrairement à la série d’Aba et al, où le siège était situé majoritairement aux membres inférieurs [3]. Le tétanos était d’emblée généralisé comme dans la plupart des séries africaines [3, 8]. La durée d’hospitalisation a été de 20 jours dans notre cas, comparable à la plupart des séries [7–9].

## Conclusion

La lutte contre le tétanos associé aux soins passe par la vaccination, l’application stricte des règles d’asepsie, et l’administration de la sérothérapie antitétanique lors de la prise en charge de toute blessure survenant chez des sujets non vaccinés ou ayant un statut immunitaire douteux. Pour ce faire une formation continue du personnel médical et paramédicalest nécessaire. Cela passe également par la mise en place d’une politique de rappel vaccinal contre le tétanos chez les grands enfants, les adultes et les personnes âgées.
